# Técnica do fulcro com lápis: Um método simples para redução fechada de fraturas da falange proximal por mecanismo de hiperabdução, o “mecanismo do pianista”, em crianças

**DOI:** 10.1055/s-0045-1813232

**Published:** 2025-12-15

**Authors:** Vivek M. Sodhai, Darshan Munot, Rahul Jaiswal, Sandeep Patwardhan

**Affiliations:** 1 Departamento de Ortopedia Pediátrica, Sancheti Institute for Orthopaedics & Rehabilitation, Pune, Índia

**Keywords:** falanges dos dedos da mão, fraturas ósseas, pediatria, redução fechada, closed fracture reduction, finger phalanges, fractures, bone, pediatrics

## Abstract

As fraturas extra-oitavas da quinta falange proximal são lesões fisárias do tipo II de Salter-Harris e provocam desvio ulnar e angulação dorsal. A redução inadequada dessas fraturas pode causar deformidade estética, perda de alinhamento e comprometimento funcional. Métodos tradicionais de redução, como a manobra de Jahss, podem ser tecnicamente complexas, agressiva e frequentemente exigem sedação ou cirurgia, o que limita seu uso em crianças pequenas e ambientes com recursos limitados.

Relatamos nossa experiência com a “técnica do lápis” assistida por fulcro para redução fechada em quatro crianças, com idades entre 4 e 11 anos, sob bloqueio nervoso digital sem sedação ou tração. O método emprega um lápis simples, que atua como fulcro, posicionado na base do quarto espaço interdigital próximo à articulação metacarpofalangiana. Uma leve pressão de adução e flexão é aplicada à falange proximal, o que corrige o desvio ulnar e a angulação dorsal por alavancagem biomecânica. A redução foi confirmada clínica e radiograficamente, realizando-se imobilização solidária com a técnica de “buddy strapping” ou tala de estanho, mantida por 3 semanas.

Na consulta de acompanhamento, realizada 6 a 12 semanas após o procedimento, todos os pacientes apresentaram redução estável, cicatrização sem intercorrências, amplitude de movimento completa e indolor, e retorno às atividades básicas. Os resultados funcionais avaliados pelo escore Quick Disabilities of the Arm, Shoulder and Hand (QuickDASH) foram excelentes: dois pacientes com pontuação 0, um com 2,27 e o último com 4,55. Não foram observadas complicações ou novas luxações.

A técnica do fulcro com lápis é simples, segura, reprodutível e econômica. Representa uma valiosa adição ao arsenal de médicos para tratamento de fraturas extra-oitavas pediátricas, particularmente em ambulatórios, emergências e instituições com poucos recursos.

## Abstract (en)

Extra-octave fractures of the fifth proximal phalanx are Salter-Harris type II physeal injuries resulting in ulnar deviation and dorsal angulation. These fractures, if inadequately reduced, may result in cosmetic deformity, malalignment, and functional impairment. Traditional reduction methods, such as the Jahss maneuver, can be technically demanding, forceful, and often require sedation or surgical assistance, which limits their use in younger children and in resource-constrained environments.

We report our experience with the fulcrum-assisted “pencil technique” for closed reduction in four children, aged 4 to 11 years, under digital nerve block without sedation or traction. The method employs a simple pencil placed at the base of the fourth web space near the metacarpophalangeal joint, serving as a fulcrum. Gentle adduction and flexion pressure is applied to the proximal phalanx, correcting both ulnar deviation and dorsal angulation by biomechanical leverage. Reduction was confirmed clinically and radiographically, and immobilization was achieved with buddy strapping or tin splinting for 3 weeks.

All patients achieved stable reduction and demonstrated uneventful healing, pain-free full range of motion, and return to baseline activities at follow-up ranging from 6 to 12 weeks. Functional outcomes assessed by the quick disabilities of the arm, shoulder, and hand (QuickDASH) scores were excellent: two patients scored 0, one 2.27, and another 4.55. No complications or redisplacements were observed.

The pencil fulcrum technique is simple, safe, reproducible, and cost-effective. It represents a valuable addition to the armamentarium of physicians managing pediatric extra-octave fractures, particularly in outpatient, emergency, and low-resource settings.

## Introdução

As fraturas pediátricas da mão estão entre as lesões mais comuns observadas em serviços de emergência e ortopedia, sendo frequentemente decorrentes de esportes, quedas ou trauma direto. Dentro desse espectro, fraturas com acometimento da base e do colo da quinta falange proximal são comuns, em especial devido à carga axial ou impacto na borda ulnar da mão. Uma variante específica dessas lesões, conhecida como “fratura extra-oitava”, é classificada como tipo II de Salter-Harris da base da quinta falange proximal. 1

**Fig. 1:**
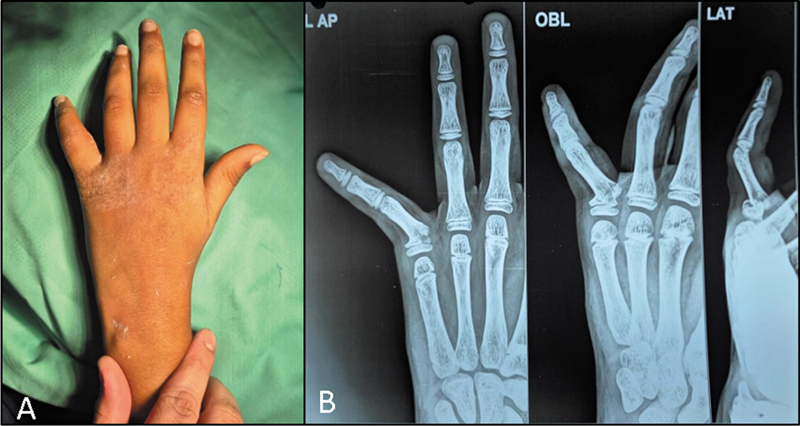
( **A** ) Imagem clínica mostrando o desvio ulnar e angulação dorsal em uma “fratura extra-oitava”. ( **B** ) Radiografia mostrando a lesão de tipo II de Salter-Harris na quinta falange proximal.

O termo “fratura extra-oitava” foi dado ao aumento do desvio ulnar e da angulação dorsal do dedo mínimo que permite uma extensão teórica da envergadura da mão semelhante a um pianista alcançando uma oitava a mais, às custas de função e alinhamento ( Fig. 1 ). 1 2

Historicamente, essas lesões eram classificadas de maneira errônea ou agrupadas com outras fraturas justaepifisárias até o melhor reconhecimento da angulação única e das implicações anatômicas da variante extra-oitava. Os primeiros relatos da literatura observaram que, embora essas fraturas parecessem inócuas, sua redução inadequada poderia causar deformidades estéticas, desalinhamento rotacional e perda do controle motor fino, particularmente em tarefas que exigem forte preensão ulnar. 1 2

O tratamento convencional dessas fraturas é a redução fechada por meio de métodos baseados em tração, como a manobra de Jahss (técnica de flexão-pressão 90-90), que foi originalmente descrita para tratamento de fraturas do colo do metacarpo e, em seguida, adaptada para lesões da base das falanges, incluindo fraturas extra-oitavas. 3 Esta manobra envolve a flexão da articulação metacarpofalangiana (AMCF) e a aplicação de força em direção volar na diáfise do metacarpo e direção dorsal na articulação interfalangiana proximal (AFP), além da imobilização para assegurar que o dígito não fique em extensão. A redução forçada com esta técnica pode agravar a lesão dos tecidos moles. Al-Qattan recomenda a imobilização com uma tala de goteira ulnar ou gesso com a AMCF flexionada em 90°. 1

Diante desses desafios, há a crescente adoção de técnicas simples, reprodutíveis e menos invasivas que possam ser realizadas com segurança em ambulatórios ou prontos-socorros. Nesse contexto, utilizamos um novo método assistido por fulcro, descrito como “método do lápis”, introduzido por Beatty et al. 4 Empregando um lápis comum ou objeto cilíndrico sólido semelhante, é feita redução fechada por meio de alavancagem biomecânica, alcançando redução anatômica dessas fraturas. 4

Apresentamos aqui uma série de quatro crianças com fraturas extra-oitavas tratadas com a redução baseada em fulcro sob bloqueio digital, usando um lápis ou objeto cilíndrico sólido semelhante, sem quaisquer instrumentos cirúrgicos.

## Métodos

Este estudo retrospectivo foi aprovado pelo Comitê de Ética Institucional e os responsáveis pelos pacientes assinaram um termo de consentimento livre e esclarecido para apresentação de dados e imagens. Conduzimos uma série retrospectiva de casos de 4 pacientes pediátricos, com idades entre 4 e 11 anos, com fraturas extra-oitavas da 5ª falange proximal entre 2023 e 2024.

Todas as fraturas foram classificadas como tipo II de Salter-Harris com acometimento da base da falange proximal. Os pacientes foram tratados no ambulatório ou no pronto-socorro usando uma técnica de redução fechada assistida por fulcro, sob bloqueio nervoso digital com lidocaína a 2%. Todos os pacientes foram avaliados quanto à cicatrização radiológica e à recuperação funcional segundo o questionário Quick Disabilities of the Arm, Shoulder and Hand (QuickDASH).

## Descrição da Técnica

**Fig. 2:**
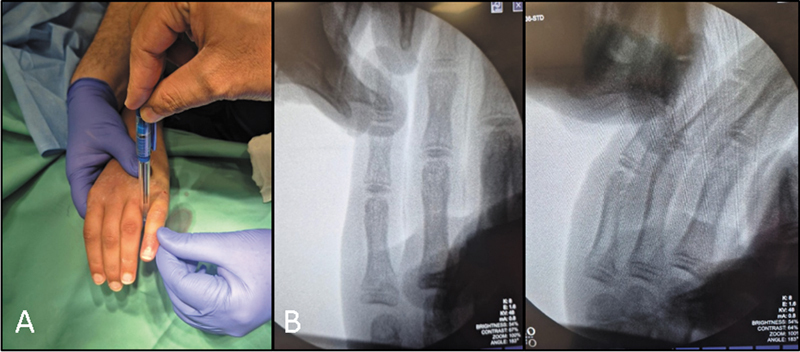
( **A** ) Demonstração da redução assistida por fulcro com lápis no quarto espaço interdigital próximo à AMCF proximal. ( **B** ) Imagem fluoroscópica intraoperatória mostrando a redução anatômica.

**Fig. 3:**
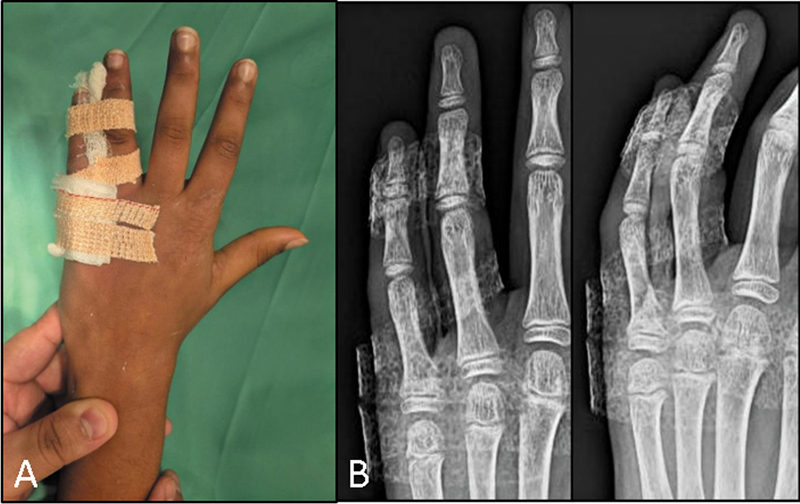
( **A** ) Imobilização solidária ( *buddy strapping* ) pós-operatória. ( **B** ) Radiografia pós-operatória mostrando a redução estável da fratura.

1. Posicionamento do paciente em decúbito dorsal com a mão acometida pronada sobre uma mesa de apoio.
2. Administração do bloqueio nervoso digital composto por lidocaína a 2% no dedo lesionado.
3. Visualização da deformidade clínica e da fratura no quinto dígito.
4. Colocação de um lápis ou um objeto cilíndrico sólido semelhante perpendicular à mesa na base do quarto espaço interdigital próximo à AMCF ( Fig. 2A ).
5. Utilizando o lápis como fulcro, aplicação de uma leve pressão para adução e flexão da falange proximal. Esse movimento coordenado facilita a redução da fratura por meio de alavancagem biomecânica, corrigindo o desvio ulnar e a angulação dorsal.
6. Confirmação da redução por métodos clínicos e radiográficos, com utilização de fluoroscopia em incidências anteroposterior, oblíqua e lateral ( Fig. 2B ).
7. Imobilização do dígito de forma solidária ( *buddy strapping* ) com ou sem tala de estanho por um período de 3 semanas ( Fig. 3A ). Radiografias foram obtidas no pós-operatório imediato ( Fig. 3B ).
8. Instituição de exercícios de amplitude de movimento ao final de 3 semanas e, em seguida, de exercícios de fortalecimento da preensão.

## Resultados

Todas as fraturas em nossa coorte ocorreram na mão direita dominante. Todos os quatro pacientes foram submetidos à redução fechada utilizando a técnica do fulcro com lápis com sucesso, sem a necessidade de tração, sedação ou instrumentação cirúrgica. A avaliação radiográfica confirmou o alinhamento anatômico em cada caso, que foi mantido até a consolidação da fratura. A imobilização foi bem tolerada.

A consolidação da fratura ocorreu sem intercorrências em todos os casos, com ausência de novas luxações, infecções ou comprometimentos neurovasculares. À última consulta de acompanhamento, 3 a 6 meses após o procedimento, todos os pacientes apresentaram amplitude de movimento completa e indolor do dígito acometido e retornaram ao nível de atividade anterior à lesão. Os resultados funcionais avaliados pelo questionário QuickDASH mostraram excelente recuperação, com pontuações de 0 em 2 casos, 2,27 em um caso e 4,55 no último ( Tabela 1 ).

**Tabela 1:** Características dos pacientes, detalhes da lesão, acompanhamento e resultados funcionais (QuickDASH)

Paciente	Idade, anos	Mão lesionada	Mão dominante	Mecanismo	Tipo de lesão	Acompanhamento	Pontuação QuickDASH
**1**	4	Esquerda	Direita	Queda sobre a mão estendida	Salter-Harris II – base da falange proximal	3 meses	4,55
**2**	11	Esquerda	Direita	Trauma direto	Salter-Harris II – base da falange proximal	6 meses	0
**3**	9	Esquerda	Direita	Lesão ao pegar uma bola de críquete	Salter-Harris II – base da falange proximal	4 meses	2,27
**4**	10	Esquerda	Direita	Trauma direto por uma bola de futebol	Salter-Harris II – base da falange proximal	6 meses	0

**Abreviatura:** QuickDASH, Quick Disabilities of the Arm, Shoulder and Hand.

## Discussão

Embora relativamente incomuns, as fraturas extra-oitavas representam um subconjunto distinto de lesões das falanges proximais pediátricas que exigem tratamento cuidadoso para prevenção de deformidades e disfunções em longo prazo. O desvio ulnar e a angulação dorsal característicos podem causar comprometimento funcional significativo em caso de redução inadequada, em especial em atividades que exigem forte preensão ulnar e controle motor fino. 1 2 Os métodos tradicionais de redução fechada, como a manobra de Jahss, 3 foram adaptados para essas lesões, mas ainda são tecnicamente complexos e desconfortáveis para crianças.

A manobra de Jahss, originalmente descrita para fraturas do colo do metacarpo, envolve a flexão de 90° da articulação do metacarpo e pressão dorsovolar oposta no sítio da fratura. Embora eficaz em mãos experientes, a técnica é inerentemente forçada e frequentemente dolorosa, com necessidade de sedação ou mesmo de anestesia geral em pacientes mais jovens. Além disso, as tentativas de redução por meio de manobras baseadas em tração podem exacerbar a lesão de tecidos moles e predispor a uma nova luxação. Estes procedimentos também têm riscos e aumentam a utilização dos serviços de saúde, incluindo o possível tempo em sala de cirurgia e a alocação de recursos. Há uma ênfase crescente no desenvolvimento de estratégias alternativas de redução que sejam minimamente invasivas e viáveis, mesmo em asilos ou clínicas em locais remotos.

Por outro lado, a técnica do fulcro com lápis oferece uma alternativa biomecanicamente simples e benéfica para o paciente. Esta técnica foi primeiramente descrita por Beatty et al. 4 Ao utilizar um objeto cilíndrico simples, como um lápis, como fulcro na base do quarto espaço interdigital, o cirurgião pode aplicar forças suaves de adução e flexão que alavancam a falange proximal até o alinhamento anatômico. Essa técnica minimiza a tração e evita a manipulação excessiva, reduzindo, assim, o desconforto e eliminando a necessidade de sedação na maioria dos casos. Nossos resultados demonstraram que a manobra é eficaz e bem tolerada sob bloqueio digital isolado.

Outros métodos de redução, como a redução percutânea com pinos ou as abordagens miniabertas, são reservados para fraturas em padrões irredutíveis ou instáveis. Embora seus resultados sejam confiáveis, essas técnicas aumentam a complexidade do procedimento e a utilização de recursos. Também apresentam riscos de infecção, lesão fisária e complicações relacionadas à anestesia. Já o método do fulcro com lápis não requer equipamento especializado, pode ser realizado com rapidez em ambulatórios ou serviços de emergência e evita a morbidade associada à intervenção cirúrgica.

Nossa pequena série corrobora a reprodutibilidade e a segurança dessa manobra. Todos os pacientes obtiveram reduções estáveis sem complicações e resultados funcionais excelentes no acompanhamento. É importante ressaltar que a ausência de novas luxações reforça a solidez biomecânica dessa abordagem assistida por fulcro em fraturas com padrões estáveis.

As limitações do nosso estudo incluem o pequeno tamanho da coorte, o acompanhamento em curto prazo e a ausência de um grupo controle comparativo. O curto acompanhamento pode ser atribuído ao menor tempo de recuperação dessas crianças. Além disso, essa técnica não é apropriada para fraturas cominutivas, expostas, extremamente instáveis ou casos com interposição de tecidos moles. Sua aplicabilidade em adolescentes ou adultos, com ossos e tecidos moles mais rígidos, ainda precisa ser determinada.

## Considerações Finais

A técnica do fulcro com lápis é um método simples, seguro, reprodutível e econômico para redução fechada de fraturas extra-oitavas em crianças. Sua mínima dependência em instrumentos especializados ou sedação a torna uma excelente opção para uso em ambulatórios, prontos-socorros e serviços com recursos limitados. Por sua reprodutibilidade e custo-efetividade, essa técnica é uma valiosa adição ao arsenal do clínico ortopédico. Estudos futuros com coortes maiores e multicêntricas e acompanhamento em longo prazo são necessários para validar ainda mais a eficácia desta técnica e otimizar as diretrizes clínicas de tratamento.

## References

[1] Szymanski S Zylstra M Hull A “One Note Higher”: A Unique Pediatric Hand Fracture Clin Pract Cases Emerg Med 2021 5 02 270 272 10.5811/cpcem.2021.3.51806 34437026 PMC8143814

[2] Mims L Khodaee M Extra-Octave Fracture in a 14-Year-Old Basketball Player J Pediatr 2017 186 206 2060 10.1016/j.jpeds.2017.03.018 28408129

[3] Jahss S A Fractures of the metacarpals: a new method of reduction and immobilization J Bone Joint Surg Am 1938 20 01 178 186

[4] Beatty E Light T R Belsole R J Ogden J A Wrist and hand skeletal injuries in children Hand Clin 1990 6 04 723 738 10.1016/S0749-0712(21)01068-4 2269682

